# Polyene Phosphatidylcholine Ameliorates High Fat Diet-Induced Non-alcoholic Fatty Liver Disease *via* Remodeling Metabolism and Inflammation

**DOI:** 10.3389/fphys.2022.810143

**Published:** 2022-02-28

**Authors:** Yang Lu, Tingting Feng, Jinxiu Zhao, Pengfei Jiang, Daxiang Xu, Menglu Zhou, Mengyu Dai, Jiacheng Wu, Fenfen Sun, Xiaoying Yang, Qisi Lin, Wei Pan

**Affiliations:** ^1^Jiangsu Key Laboratory of Immunity and Metabolism, Department of Pathogenic Biology and Immunology, Xuzhou Medical University, Xuzhou, China; ^2^First Clinical Medicine College, Xuzhou Medical University, Xuzhou, China; ^3^Jiangsu Key Laboratory of New Drug Research and Clinical Pharmacy, Xuzhou Medical University, Xuzhou, China; ^4^Department of Pharmacy, The First Affiliated Hospital of Henan University of Science and Technology, Luoyang, China; ^5^Second Clinical Medicine College, Xuzhou Medical University, Xuzhou, China

**Keywords:** polyene phosphatidylcholine, non-alcoholic fatty liver disease, liver steatosis, high fat diet, metabolic remodeling, inflammation

## Abstract

Recent years have witnessed a rise in the morbidity of non-alcoholic fatty liver disease (NAFLD), in line with the global outbreak of obesity. However, effective intervention strategy against NAFLD is still unavailable. The present study sought to investigate the effect and mechanism of polyene phosphatidylcholine (PPC), a classic hepatoprotective drug, on NAFLD induced by high fat diet (HFD). We found that PPC intervention reduced the mass of liver, subcutaneous, epididymal, and brown fats in HFD mice. Furthermore, PPC supplementation significantly mitigated liver steatosis and improved glucose tolerance and insulin sensitivity in HFD mice, which was accompanied by declined levels of hepatic triglyceride, serum triglyceride, low density lipoprotein, aspartate aminotransferase, and alanine aminotransferase. Using transcriptome analysis, there were 1,789 differentially expressed genes (| fold change | ≥ 2, *P* < 0.05) including 893 upregulated genes and 896 downregulated genes in the HFD group compared to LC group. A total of 1,114 upregulated genes and 1,337 downregulated genes in HFD + PPC group were identified in comparison to HFD group. With the help of Gene Ontology (GO) analysis, these differentially expressed genes between HFD+PPC and HFD group were discovered related to “lipid metabolic process (GO: 0006629),” “lipid modification (GO: 0030258),” and “lipid homeostasis (GO: 0055088)”. Though Kyoto Encyclopedia of Genes and Genomes (KEGG) pathway analysis, we found pathways associated with hepatic homeostasis of metabolism and inflammation. Notably, the pathway “Non-alcoholic fatty liver disease (mmu04932)” (*P*-value = 0.00698) was authenticated in the study, which may inspire the potential mechanism of PPC to ameliorate NAFLD. The study also found that lipolysis, fatty acid oxidation, and lipid export associated genes were upregulated, while the genes in uptake of lipids and cholesterol synthesis were downregulated in the liver of HFD mice after PPC supplementation. Interestingly, PPC attenuated the metabolic inflammation *via* inhibiting pro-inflammatory macrophage in the livers of mice fed by HFD. In summary, this study demonstrates that PPC can ameliorate HFD-induced liver steatosis *via* reprogramming metabolic and inflammatory processes, which inspire clues for further clarifying the intervention mechanism of PPC against NAFLD.

## Introduction

Abnormal lipid deposition in the liver leads to non-alcoholic fatty liver disease (NAFLD), which encompasses a spectrum of liver disorders ranging from hepatic steatosis to non-alcoholic steatohepatitis and ultimately may lead to cirrhosis (Manne et al., 2018). As the most common chronic liver disease worldwide, NAFLD affects 25% of the global adult population (Younossi et al., 2019) and its prevalence is expected to increase rapidly soon owing to the global epidemics of obesity and type 2 diabetes (Park et al., 2020). It is now increasingly clear that NAFLD not only affects the liver but can also increase the risk of developing extra-hepatic diseases, including metabolic syndrome (Dietrich and Hellerbrand, 2014; Yki-Järvinen, 2014), cardiovascular disease (Kim et al., 2012), and chronic kidney disease (Musso et al., 2015), placing a heavy burden on health-care resources. However, the effective intervention strategy against NAFLD is still unavailable.

The progression of NAFLD involves multifactorial events. In the well-recognized “two-hit model” theory, the lipids secondary to diet or genetic factors-induced insulin resistance is first deposited in livers (Buzzetti et al., 2016), and then chronic lipid exposure destroys the lysosomal-mitochondrial axis and erupts intracellular reactive oxygen species overproduction, stimulating lipid peroxidation and inflammatory cascades (Ruhl and Everhart, 2004; Brunt, 2010; Peverill et al., 2014). Thus, the rebuilding of hepatic lipid metabolism homeostasis can effectively prevent NAFLD (Seebacher et al., 2020). Moreover, there are numerous studies showing that proinflammatory cascades are also central to NAFLD progression (Krenkel and Tacke, 2017). It is reported that pro-inflammatory macrophages, also known as Kupffer cells (KC), can stimulate or dismantle liver fibrosis by secreting cytokines such as tumor necrosis factor-α (TNF-a) and interleukin-1β (IL-1β) in the liver (Huang et al., 2010). In contrast, the M2 KCs have pro-apoptotic effects on KCs (Wan et al., 2014) and promote the resolution of inflammation *via* IL-10, thereby protecting hepatocytes against NAFLD (Han et al., 2017). Taken together, reprogramming hepatic metabolism and inflammation provides an attractive strategy for NAFLD intervention.

Polyene phosphatidylcholine (PPC), a major active ingredient of essential phospholipids, plays a key role in maintaining membrane fluidity and function. In 2000, PPC has already attracted attention for its protection against liver injury (Mi et al., 2000). Later, PPC is reported to improve the NAFLD and repair damaged hepatic cell membrane (Okiyama et al., 2009). PPC could alleviate liver injury and promote liver function restoration by recovering oxidative balance and improving inflammation (Cao et al., 2016). It is worth mentioning that a prospective study carried out in Russia found PPC as an adjunctive therapy may be useful in improving the ultrasonographic features of NAFLD in patients with associated cardiometabolic comorbidities (Maev et al., 2020). Moreover, our previous study showed a protective effect of PPC on a collagen-induced arthritis model in rats (Pan et al., 2017). In addition, our recent study revealed that PPC interacting with TLR-2 reprograms the lipid metabolism to alleviate the inflammatory response triggered by LPS in macrophages (Feng et al., 2020). Overall, these findings indicate that PPC is a potential drug against inflammatory and metabolic diseases. However, it is still blurry if PPC could improve NAFLD *via* remodeling inflammation and metabolism.

The present study investigated the effect of PPC on NAFLD induced by high fat diet (HFD) and explored the potential mechanism. The results showed that PPC supplementation reduces liver weight and fat mass, improves insulin resistance in mice fed by HFD. Notably, the liver steatosis was significantly alleviated after PPC treatment. Furthermore, several differentially expressed genes and related metabolic events were implicated in the beneficial effect of PPC on the improvement of NAFLD. Overall, this study reveals PPC can prevent NAFLD, which is associated with the reconstitution of liver metabolic and inflammatory profiles.

## Materials and Methods

### Animal Groups and Polyene Phosphatidylcholine Intervention

Male C57BL/6J mice (8 weeks old) were obtained from the Laboratory Animal Center of Xuzhou Medical University (Xuzhou, China) and were bred in the specific pathogen-free facilities. The mice were randomly divided into four groups (*n* = 8 for each group): (1) mice fed a low-fat diet (5% fat by weight) and intraperitoneally injected with 200 μl 10% glucose (twice per week) as the LC group; (2) mice fed a low-fat diet (5% fat by weight) and intraperitoneally injected with 200 μl 10% glucose solution containing 20 μg PPC (twice per week) as the PPC group; (3) mice fed a high fat diet (60% fat by weight) and intraperitoneally injected with 200 μl 10% glucose (twice per week) as the HFD group; and (4) mice fed a high fat diet (60% fat by weight) and intraperitoneally injected with 200 μl 10% glucose solution containing 20 μg PPC (twice per week) as the HFD + PPC group. The PPC injection was purchased from Tiantai Mount Pharmaceutical Co., Ltd. (Chengdu, China). The concentration of PPC was calculated at the basis of our previous study (Pan et al., 2017). The high fat purified rodent diet was purchased from Dyets (article number: HF60) and the diet contains 20 kcal% protein, 20 kcal% carbohydrate, and 60 kcal% fat. All mice were housed in standard laboratory conditions at 22 ± 2°C and relative humidity 55 ± 10% with 12 h dark-light cycle and permitted regular unrestricted food and water. All mice were sacrificed for experiment at 13th week. Before sacrifice, no abnormal mortality of mice was observed. After sacrifice, liver and adipose tissue (epididymal, subcutaneous and brown) were weighed and collected for further processing and analyses. The surgery was performed following a previous protocol (Bagchi and MacDougald, 2019).

The sample size was determined by power analysis on website^1^ at the liberal significance level of α = 0.05 (two-sided) and the power of 1-β = 80%. The sample size *n* = 8 each group was estimated based on the data of serum TG, AST and ALT level in the previous intervention (Lv et al., 2019): mean 1 = 1.2, mean 2 = 3.4, *SD* = 0.9, power of 1-β = 0.9994 in serum TG level; mean 1 = 13, mean 2 = 42, *SD* = 14, power of 1-β = 0.9917 in serum AST level; mean 1 = 16, mean 2 = 52, *SD* = 20, power of 1-β = 0.9631 in serum ALT level.

### The Intraperitoneal Glucose Tolerance Test

On the day before the Intraperitoneal Glucose Tolerance Test (IPGTT), mice were fast overnight (16 h), while ensuring that the mice have access to drinking water. Each mouse was then received an intraperitoneal injection of glucose (2 g/kg body weight, Sigma-Aldrich, United Kingdom). Blood samples were collected from the tail and measured with blood glucose test strips at 0, 30, 60, 90, and 120 min (Nagy and Einwallner, 2018). The curve of blood glucose over time was drawn by GraphPad 8.0 software and the total area under the curve (AUC) was calculated using the trapezoidal rule.

### Homeostasis Model Assessment Score Analysis

After the mice were fasted for 6 h, the fasting blood glucose levels of the mice were detected according to the determination method in IPGTT, and an appropriate amount of whole blood was collected by squeezing the tail vein of mouse. Blood samples were coagulated for 1 h at room temperature and centrifuged at 12,000 rpm/min for 15 min. Subsequently, the insulin levels were determined according to the Mouse Insulin Ultra-Sensitive ELISA kit (Crystal Chem, United States). The homeostasis model assessment-insulin resistance (HOMA-IR) was applied to estimate the insulin sensitivity (Matthews et al., 1985), and was calculated according to the formula (HOMA-IR = blood glucose concentration × insulin concentration/22.5).

### Biochemical Indices

After centrifuged at 12,000 rpm, 4°C for 20 min, the eyeball serum concentration of aspartate aminotransferase (AST), alanine aminotransferase (ALT), triglyceride (TG), high-density lipoprotein (HDL), and low-density lipoprotein (LDL) was measured by the automatic biochemical analyzer (Roche Cobas 701, Roche Diagnostics Ltd.). The hepatic TGs were determined by using the Triglyceride Assay Kit from Nanjing Jiancheng Bioengineering Institute (A110-1-1) following manufacturer’s instructions.

### Histopathological Examination

Histopathological Examination Liver tissues were stained with Hematoxylin and eosin (H&E) according to the protocol (Fischer et al., 2008; Cardiff et al., 2014) to show the pathological changes. First, slices were put into the xylene for 20 min to get dewaxed. Then, we put slices into anhydrous ethanol, 95% ethanol and 70% ethanol for 2 min, respectively, to hydrate the samples. After that, the tissues were stained with hematoxylin solution for 3 min and were then rinsed under running tap until the water was colorless. Next, the tissues were stained with eosin Y solution for 2 min. Finally, dehydrate the samples, clear the samples with xylene and add a coverslip.

Oil red staining of liver tissue was performed to assess the amount of fat drops. After drying at room temperature, sections were impregnated into oil red solution for 8–10 min. The sections were then put into the two cylinders containing 60% isopropyl alcohol for 3 and 5 s, respectively. Sections were washed three times with distilled water and photographed with a microscope.

### Immunohistochemical Staining

Liver tissue was fixed in 10% normal buffered formalin overnight and embedded in paraffin wax. Five-micrometer-thick sections were used. The sections were deparaffinized and then boiled at 95°C for 20 min in sodium citrate solution for antigen retrieval. To assess for macrophage activity, rabbit antibody to macrophage biomarkers including F4/80, CD206 and CD11c (Servicebio Technology Co., Ltd., Wuhan, China) was used. The sections were incubated overnight at 4°C at a dilution of 1:1,000. A standard avidin-biotin complex method (Vector Laboratories) was used for the secondary antibody (anti-rabbit), using a 1:200 dilution and a 1-h incubation. Slides were developed using a peroxidase detection kit counterstained with hematoxylin after immunolabeling.

Quantification of positively stained cells was carried out by imageJ following official instructions.^2^ First, images were transferred into 8-bit black-and-white images. Then, a threshold range was set manually to tell the positive cells apart from the background. At last, we used the plugin named “Nucleus Counter” to get information about number of positive cells in the image.

### RNA Library Construction and Sequencing

Liver samples of three LC, PPC, HFD, and HFD + PPC mice were detected. The transcriptome analysis (RNA-seq) was conducted in CapitalBio Technology Co., Ltd. For mRNA library construction and deep sequencing, RNA samples were prepared by using the TruSeq RNA Sample Preparation Kit according to the manufacture’s protocol. Briefly, the poly-A containing mRNA molecules were purified from the 3 μg of total RNA by using poly-T oligo-attached magnetic beads. The cleaved RNA fragments were reversely transcribed into first-strand cDNA using random hexamers, following by second-strand cDNA synthesis using DNA Polymerase I and RNase H. The cDNA fragments were purified, end blunted, “A” tailed, and adaptor ligated. PCR was used to selectively enrich those DNA fragments that have adapter molecules on both ends and to amplify the amount of DNA in the library. The number of PCR cycles was minimized (12 cycles) to avoid skewing the representation of the library. The library was qualified by Agilent 2,100 bioanalyzer and quantified by Qubit and qPCR. The produced libraries were sequenced on the HiSeq 2,500 platform.

### Differential Expression Analysis of Transcriptional Profiling

According to credibility interval approaches reported for the analysis of SAGE data (Robinson and Smyth, 2008), the edgeR (Robinson et al., 2010) program was used to identify differentially expressed mRNAs based on their relative quantities which were reflected by individual gene reads (Robinson and Oshlack, 2010). The method uses empirical Bayes estimation and exact tests based on the negative binomial distribution. Genes with a *P*-value ≤ 0.05 and expression ratio ≥ 2 or expression ratio ≤ 0.5 were recognized as significantly differentially expressed genes between the two samples.

### Gene Ontology and Kyoto Encyclopedia of Genes and Genomes Enrichment Analysis

The functional assignments were mapped onto Gene Ontology (GO). Biological process, cellular component, and molecular function were involved in the GO terms. The enrichment analysis of GO terms with *P* < 0.05 was considered significantly changed between two groups. Moreover, genes were compared with the Kyoto Encyclopedia of Genes and Genomes (KEGG) pathway analysis by using BLASTX (Altschul et al., 1997) at *E*-values ≤ 1e-10. Then, a Perl script program was used to retrieve KO information from the blast results and associations between genes and pathways were established.

### Clustering Analysis

Both total genes and differentially expressed genes were used to generate clustering diagrams by Cluster 3.08 with the hierarchical method. The uncentered correlation and average linkage parameters were chosen to calculate gene distance and sample distance. Meanwhile, genes related to metabolism and inflammation were picked ulteriorly to generate clustering diagrams by the R programming language.

### Quantitative Reverse Transcription PCR

Quantitative reverse transcription-PCR (qRT-PCR) was performed to detect the mRNA expression of the genes related to fatty acid synthesis, fatty acid oxidation, and inflammation. Total RNA was extracted with TRIzol from liver tissues and 1 μg RNA for each sample was firstly reverse-transcripted to cDNA. Real-time PCR was performed using LightCycler^®^ 480 II Real-time PCR Instrument (Roche, Swiss) with 10 μl PCR reaction mixture. The exact thermal cycler conditions were described in a previous study (Li et al., 2016). Each sample was run in triplicate for analysis. The primer sequences are shown in Supplementary Table 1. The expression levels were calculated using the 2^–ΔΔ*Ct*^ method (Livak and Schmittgen, 2001).

### Statistical Analysis

All statistical data in the study was analyzed using the software GraphPad Prism 8.0 and was presented as the mean with standard error of the mean (SEM). Statistical significance was determined using the one-way analysis of variance (ANOVA) followed by the *post hoc* Tukey test for multiple comparisons. *P* < 0.05 was considered as statistically significant.

## Results

### Polyene Phosphatidylcholine Improves Metabolic Parameters in the Mice Fed by High Fat Diet

**FIGURE 1:**
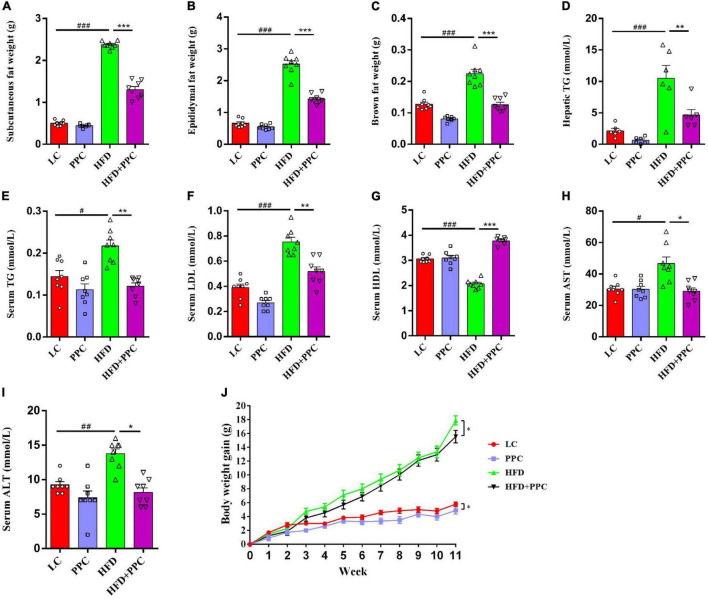
PPC supplementation improves metabolic parameters in the mice fed by HFD. All the mice were sacrificed at 13th week. **(A–C)** The mass of subcutaneous fat, epididymal fat, and brown fat. **(D)** The hepatic TG level. **(E–I)** The serum levels of TG, LDL, HDL, AST and ALT. **(J)** The body weight gain. *n* = 8 mice for each group. The differences were analyzed using one-way or two-way ANOVA. Data represent means with SEM. The pound signs indicate statistically significant differences compared to the LC group. ^#^*P* < 0.05, ^##^*P* < 0.01, ^###^*P* < 0.001. Asterisks indicate statistically significant differences compared to the HFD group. *P < 0.05, **P < 0.01, ***P < 0.001.

This study firstly evaluated the effect of PPC supplementation on HFD-induced metabolic disorder. As shown in Figure 1, a decline in mass of subcutaneous, epididymal, and brown fats was observed in HFD mice following PPC supplementation (All *P* < 0.001, Figures 1A–C). Furthermore, HFD fed mice showed elevated levels of TG in livers, TG, LDL, AST and ALT in sera, while the level of serum HDL was significantly declined (*P* < 0.05, Figures 1D–I). On the contrary, PPC supplementation reversed these changes (*P* < 0.05, Figures 1D–I). In addition, the body weight gain was monitored every week, and PPC supplementation significantly decreased the cumulative weight gain of HFD fed mice (*P* < 0.05, Figure 1J). Taken together, these results suggested PPC prevents HFD-induced obesity and attenuates the metabolic disorder.

### Polyene Phosphatidylcholine Mitigates Liver Steatosis in the Mice Fed by High Fat Diet

**FIGURE 2:**
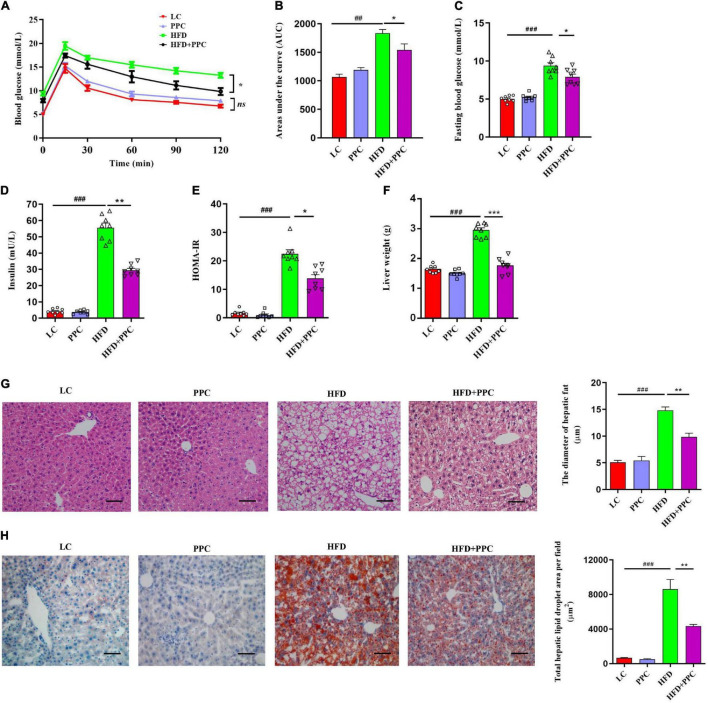
PPC supplementation improves insulin sensitivity and alleviates liver steatosis in the mice fed by HFD. **(A)** Glycemia changes in every 30 min. **(B)** AUC for GTT. **(C)** Fasting glycemia. **(D)** Blood insulin. **(E)** HOMA-IR. **(F)** The mass of liver. **(G)** Representative liver images and statistical analysis of H&E staining. **(H)** Representative liver images and statistical analysis of oil red staining. The scale bar is 50 μm. *n* = 8 mice for each group. The differences were analyzed using ANOVA. Data represent means with SEM. The pound signs indicate statistically significant differences compared to the LC group. ^#^*P* < 0.05, ^##^*P* < 0.01, ^###^*P* < 0.001. Asterisks indicate statistically significant differences compared to the HFD group. *P < 0.05, **P < 0.01, ***P < 0.001. ns, no significance.

Considering the protective effects of PPC against obesity, we assumed that PPC supplementation may defend mice against HFD-induced hepatic steatosis. H&E and Oil red staining showed the excessive accumulation of lipid droplets in the liver of HFD fed mice (Figures 2G,H). However, PPC supplementation obviously decreased the number of lipid droplets (Figures 2G,H). In the glucose tolerance test, PPC administration significantly reduced the fasting concentration of blood glucose and obviously improved the glucose tolerance sensitivity in HFD mice (*P* < 0.001, Figures 2A–C). The result of HOMA-IR showed that insulin sensitivity of HFD fed mice was also improved after PPC supplementation (*P* < 0.05, Figures 2D,E). What’s more, PPC decreased liver weight of HFD mice remarkably (*P* < 0.001, Figure 2F). These results indicated that PPC ameliorates the NAFLD induced by HFD.

### Polyene Phosphatidylcholine Alters the Transcriptome Profile in the Livers of Mice Fed by High Fat Diet

**FIGURE 3:**
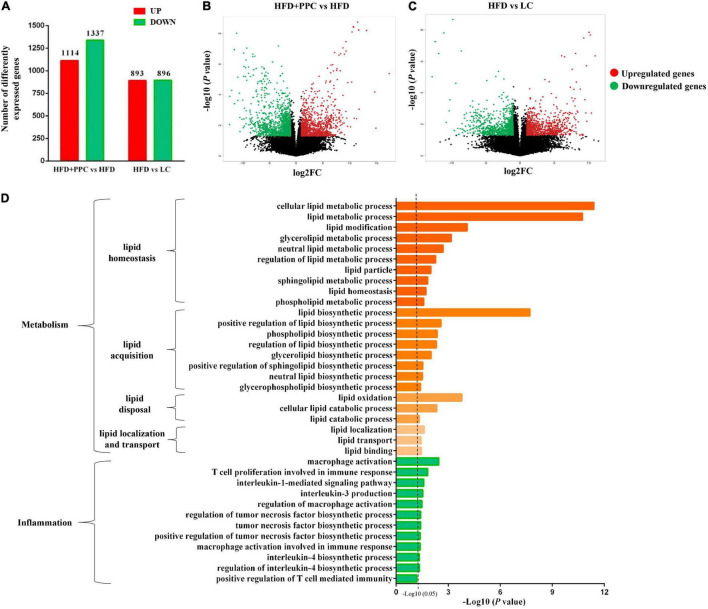
PPC supplementation remodels the transcriptome profile of liver in the mice fed by HFD. **(A)** The number of DEGs. **(B)** The volcano plot shows the distributions of DEGs between HFD + PPC and HFD mice. **(C)** The volcano plot of DEGs between HFD and LC. The *x*-axis indicates the fold change. red dots, upregulated; green dots, downregulated. **(D)** Go annotation of DEGs between HFD + PPC and HFD with terms related to hepatic metabolism and inflammation. *n* = 3 for each group.

Transcriptome analysis of liver tissues from the four groups was performed to identify how PPC improves NAFLD. The heatmap showed the correlation among different groups (Supplementary Figure 1A). Differentially expressed genes (DEGs) were screened after filtering the raw data according to *P*-value < 0.05 and expression ratio ≥ 2 or expression ratio ≤ 0.5. In compared to LC group, there were 1789 DEGs (including 893 upregulated genes, and 896 downregulated genes) in the HFD group (Figure 3A). Meanwhile, 1,114 upregulated genes and 1,337 downregulated genes in HFD + PPC group were identified in comparison to HFD group (Figure 3A). The volcano plots of DEGs were shown in Figures 3B,C, respectively. The Venn diagrams of DEGs, upregulated genes and downregulated genes were shown in Supplementary Figures 1B–D, respectively.

The DEGs between HFD + PPC and HFD groups were classified into three different functional categories including biological process, cellular component and molecular function based on the Gene Ontology (GO) analysis. Supplementary Figure 2 shows the GO annotation with the top 30 enrichment scores of the three categories, respectively. In this study, biological processes related to lipid metabolism were specially identified, including “cellular lipid metabolic process (GO: 0044255),” “lipid modification (GO:0030258),” “lipid metabolic process (GO: 0006629),” “lipid biosynthetic process (GO: 0008610)” and “lipid oxidation (GO: 0034440)” (Figure 3D). The shift of these terms suggested that PPC supplementation rebuilds lipid metabolic homeostasis and thereby improves HFD induced liver steatosis. Moreover, the shift of terms associated with inflammation revealed the effect on ameliorating inflammation of PPC (Figure 3D).

**FIGURE 4:**
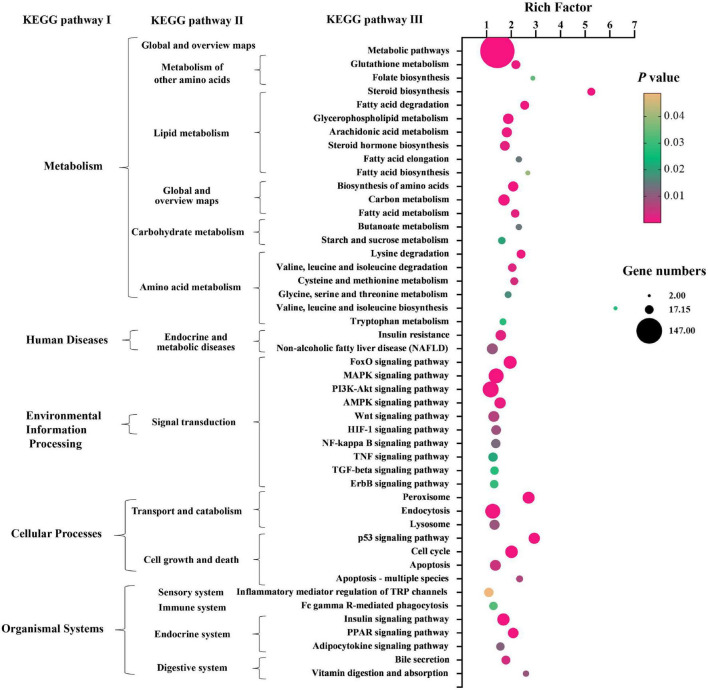
PPC supplementation modulates KEGG pathways in HFD mice. The bubble chart shows the dysregulated terms in three KEGG levels between HFD + PPC and HFD mice (*P* < 0.05). The larger the point, the more genes fall into this pathway and the yellower point means the higher significance of enrichment.

**FIGURE 5:**
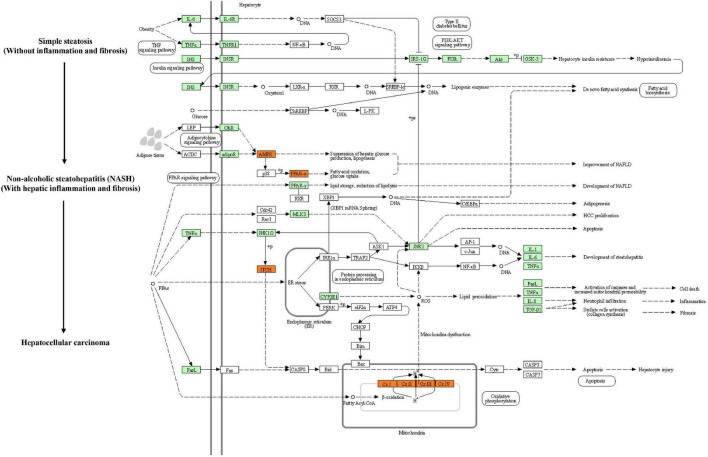
PPC improves NAFLD by regulating hepatic metabolism and inflammation in HFD mice. The flow chart shows the Non-alcoholic fatty liver disease pathway (mmu04932) in KEGG. Genes identified as DEGs between HFD and HFD + PPC in our study are marked in red frames (upregulated) and green frames (downregulated), while others are marked in blank frames.

Kyoto Encyclopedia of Genes and Genomes (KEGG) pathway enrichment analysis was performed to further understand the biological pathway of the aforementioned DEGs between HFD + PPC and HFD mice. The top 30 enriched pathways according to *P*-value were shown in Supplementary Figure 3A. Pathways including “Metabolic pathways (mmu01100)”, “Insulin signaling pathway (mmu04910)”, “PPAR signaling pathway (mmu03320)”, “Fatty acid degradation (mmu00071)”, “Carbon metabolism (mmu01200)”, “Glutathione metabolism (mmu00480)”, “Fatty acid metabolism (mmu01212)” and “PI3K-Akt signaling pathway (mmu041151)” were mapped (Figure 4), which were thought to be closely associated with the protective effect of PPC in the improvement of NAFLD. In particular, the dysregulated KEGG pathway of “Non-alcoholic fatty liver disease (mmu04932)” intrigued us. In view of this pathway, several DEGs were identified including interleukin 6 (Il6), tumor necrosis factor (Tnf), insulin receptor isoform X1 (Insr), phosphatidylinositol 3-kinase regulatory subunit (Pi3k), peroxisome proliferator-activated receptor alpha (Ppar-α), and 5′-AMP-activated protein kinase (Ampk) (Figure 5). These genes suggest the potential therapeutic targets of PPC for exerting anti-NAFLD effects and deserve further study.

### Polyene Phosphatidylcholine Enhances the Lipid Metabolism in the Liver of Mice Fed by High Fat Diet

**FIGURE 6:**
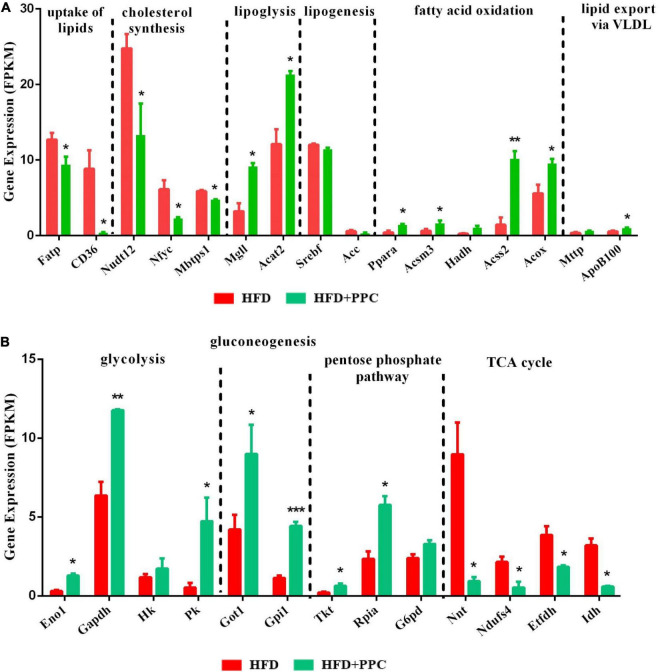
PPC supplementation reprograms the glucose and lipid metabolism in the liver of mice fed by HFD. **(A)** The expression of genes in lipid metabolism. **(B)** The expression of genes in glucose metabolism. *n* = 3 for each group. The differences were analyzed using two-tailed Student’s *t*-test. Data represent means with SEM. Asterisks indicate statistically significant differences compared to the HFD group. *P < 0.05, **P < 0.01, ***P < 0.001.

Apart from the mentioned DEGs in pathway mmu04932, there may be other genes regulating lipid metabolism in the liver of mice fed by HFD. To further characterize the specific events in the hepatic metabolism mediated by PPC supplementation, we analyzed the expression of several key genes that have been demonstrated to master the lipid metabolic pathways. As shown in Figure 6A, after PPC intervention, genes related to uptake of lipids (fatty acid transport proteins, Fatp; cluster of differentiation 36, CD36) and cholesterol synthesis (peroxisomal NADH pyrophosphatase 12, Nudt12; nuclear transcription factor-Y gamma, Nfyc; membrane-bound transcription factor site-1 protease, Mbtps1) were downregulated, while the expression of genes in lipolysis (monoglyceride lipase, Mgll; acetyl-Coenzyme A acetyltransferase 2, Acat2), fatty acid oxidation (peroxisome proliferator-activated receptor alpha, Ppara; mitochondrial Acyl-coenzyme A synthetase, Acsm3; hydroxyacyl-coenzyme A dehydrogenase, Hadh; cytoplasmic Acetyl-coenzyme A synthetase, Acss2; peroxisomal acyl-coenzyme A oxidase, Acox) and lipid export (microsomal triglyceride transfer protein, Mttp; apolipoprotein B-100, Apob100) were significantly upregulated in the HFD mice (*P* < 0.05). Additionally, lipogenic genes including sterol regulatory element-binding transcription factor 1 (Srebf1) and acetyl-CoA carboxylase (Acc) were not changed (Figure 6A). Collectively, the results suggested that PPC could alter hepatic lipid deposition mostly by enhancing lipid disposal. Furthermore, PPC activated the expression of genes involved in glycolysis, gluconeogenesis and pentose phosphate protuberance, along with suppression of tricarboxylic acid cycle (Figure 6B). These results indicated that PPC can improve hepatic metabolic disorder induced by HFD mice.

To compare the effect of PPC in the absence of HFD, we compared the transcriptomic profiles of LC and PPC groups. After filtering the raw data according to *P*-value < 0.05 and expression ratio ≥ 2 or expression ratio ≤ 0.5, there were 599 upregulated genes and 904 downregulated genes between the two groups (Supplementary Figures 5A,B). Moreover, biological processes related to lipid metabolism were specially identified by GO analysis (Supplementary Figure 5C). In addition, KEGG enrichment analysis showed the regulatory effect of PPC on the lipid metabolism and inflammatory response (Supplementary Figure 6A). Interestingly, PPC significantly upregulated several genes related to fatty acid oxidation and anti-inflammatory macrophage in LC mice (Supplementary Figure 6B). Thus, PPC still has a potential in improving hepatic immunity and metabolism in low-fat diet-fed mice.

### Polyene Phosphatidylcholine Attenuates the Metabolic Inflammation *via* Inhibiting Pro-inflammatory Macrophage Polarization in the Liver of Mice Fed by High Fat Diet

**FIGURE 7:**
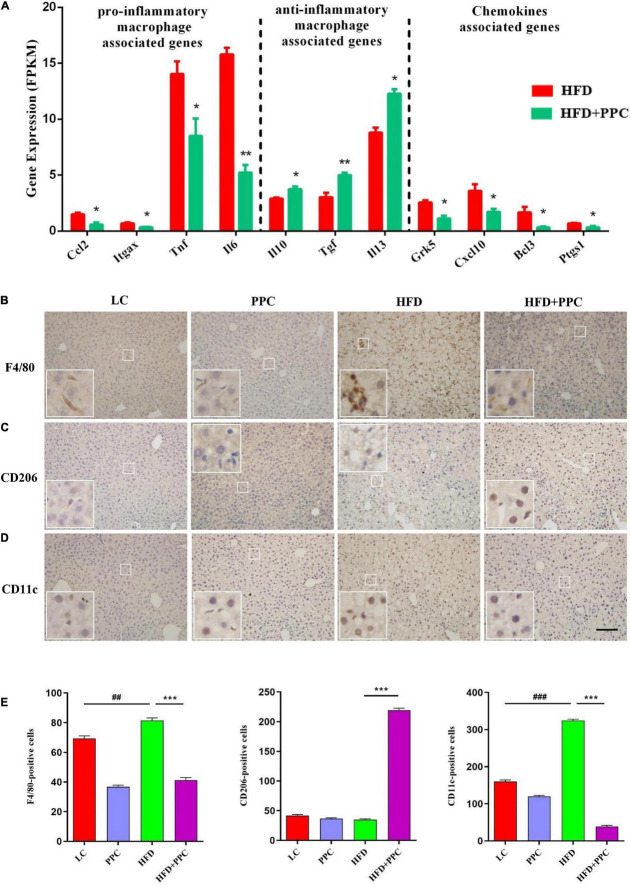
PPC supplementation attenuated the metabolic inflammation *via* inhibiting pro-inflammatory macrophage polarization in the liver of mice fed by HFD. **(A)** The expression of genes related to pro-inflammatory macrophage, anti-inflammatory macrophage, and chemokines. *n* = 3 for each group. **(B)** Immunohistochemical analysis of F4/80 positive cells in liver. **(C)** Immunohistochemical analysis of CD206 positive cells in liver. **(D)** Immunohistochemical analysis of CD11c positive cells in liver. **(E)** Counting of positive cells *via* ImageJ. Statistical significance was determined using the ANOVA followed by the *post hoc* Tukey test for comparisons. Two-tailed Student’s *t*-test was used for comparison between two groups. Data represent means with SEM. The pound signs indicate statistically significant differences compared to the LC group. ^##^*P* < 0.01, ^###^*P* < 0.001. Asterisks indicate statistically significant differences compared to the HFD group. *P < 0.05, **P < 0.01, ***P < 0.001. The scaleplate of the representative images is 50 μm.

It is increasingly recognized that pro-inflammatory macrophages contribute to the progression of NAFLD (Krenkel and Tacke, 2017). To determine whether PPC has a therapeutic effect on the hepatic inflammation in HFD fed mice, we investigated the expression of macrophage polarization associated markers based on the transcriptome analysis. As shown in Figure 7A, the expression levels of pro-inflammatory macrophages associated genes (C-C motif chemokine 2, Ccl2; integrin alpha-X, Itgax; tumor necrosis factor, Tnf; interleukin-6, Il6) of HFD + PPC mice were significantly downregulated in compared to HFD mice, while the expression of anti-inflammatory type macrophages associated genes (interleukin-10, Il10; transforming growth factor, Tgf; interleukin-13, Il13) were obviously upregulated (*P* < 0.05). Moreover, PPC supplementation significantly downregulated the expression of chemokine associated genes (G protein-coupled receptor kinase 5, Grk5; C-X-C motif chemokine 10, Cxcl10; B-cell lymphoma 3, Bcl3; prostaglandin-endoperoxide synthase 1, Ptgs1) in HFD fed mice (*P* < 0.05). Immunohistochemical analysis showed that the number of F4/80^+^ and CD11c^+^ macrophages was significantly increased in the liver of HFD mice in compared to that in LC diet mice, and there was a similar number of CD206^+^ cells between the two groups (Figures 7B–E). However, PPC supplementation prevented those changes in the HFD mice, which was characterized by an obvious numbered increase of CD206^+^ cells (Figure 7C). These results indicated that PPC alleviates the metabolic inflammation in the liver of HFD mice.

### Polyene Phosphatidylcholine Enhances Lipolysis and Alleviates Inflammation in the Fat Tissues of Mice Fed by High Fat Diet

**FIGURE 8:**
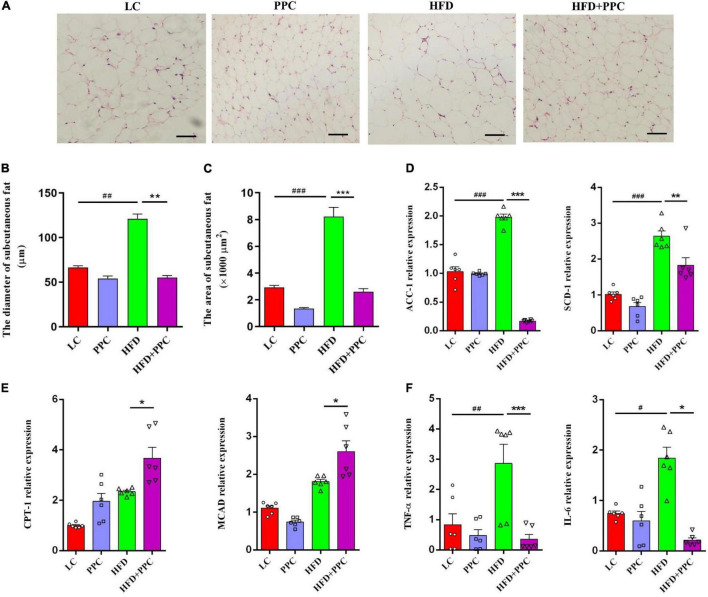
PPC supplementation enhances lipolysis and alleviates inflammation in the subcutaneous adipose tissues of mice fed by HFD. **(A)** Representative adipose tissue images of H&E staining. **(B)** The statistical results of subcutaneous adipocyte diameter. **(C)** The statistical results of subcutaneous adipocyte superficial area. **(D)** The expression of key enzymes in synthesis of fatty acids. **(E)** The expression of key enzymes related to fatty acid oxidation. **(F)** The expression of genes related to inflammation. The scaleplate of the representative images is 50 μm. *n* = 6 for each group. The differences were analyzed using ANOVA. Data represent means with SEM. The pound signs indicate statistically significant differences compared to the LC group.^#^*P* < 0.05, ^##^*P* < 0.01, ^###^*P* < 0.001. Asterisks indicate statistically significant differences compared to the HFD group. *P < 0.05, **P < 0.01, ***P < 0.001.

In obese individuals, adipose tissue can no longer effectively store lipid (adipose expandability) (Virtue and Vidal-Puig, 2010), thus redirecting lipids toward other organs, most notably the liver (Azzu et al., 2020), leading to NAFLD progression. Therefore, lipolysis enhancement of adipose tissues may ameliorate NAFLD. H&E staining showed the shrunken adipocytes in HFD + PPC subcutaneous fat tissue compared with HFD group (Figure 8A). In line with this, the diameter and superficial area of adipocytes were decreased in the HFD mice following PPC supplementation (*P* < 0.001, Figures 8B,C). Moreover, the PPC administration significantly inhibited the mRNA expression of the genes related with fatty acid synthesis (Acetyl-CoA Carboxylase 1, ACC1; Stearoyl-CoA Desaturase 1, SCD1) in the HFD mice (Figure 8D), meanwhile the expression of key enzymes associated with fatty acid oxidation such as Carnitine Palmitoyl Transferase 1 (CPT1) and Medium-Chain acyl-CoA Dehydrogenase (MCAD) were obviously upregulated (Figure 8E). Furthermore, the mRNA expression levels of interleukin-6 (IL-6) and tumor necrosis factor-α (TNF-α) in HFD mice were significantly upregulated (Figure 8F). However, PPC treatment inhibited the upregulated expression of these proinflammatory cytokines (Figure 8F). Furthermore, those alternations also occurred in the epididymal fat in the HFD mice after PPC treatment (Supplementary Figure 4). Overall, these results showed that PPC supplementation enhances lipolysis and alleviates inflammation in the fat tissues of mice fed by HFD.

## Discussion

The present study, with an obese induced NAFLD model, demonstrated the beneficial effects of PPC supplementation on the hepatic glycolipid metabolic disorder, metabolic inflammation, and improvement of liver steatosis. We showed that PPC supplementation alleviated the hepatic lipidosis induced by the HFD. Notably, the transcriptome analysis showed that PPC supplementation significantly repressed the uptake of lipids, lowered the cholesterol synthesis, and enhanced the lipolysis, fatty acid oxidation, and lipid export in HFD fed mice, which was in line with the improved hepatic pathology. Furthermore, PPC supplementation significantly mitigated macrophage inflammation in the liver of HFD mice. Collectively, these data demonstrated that PPC ameliorates the liver steatosis induced by chronic HFD, and these liver-protective effects potentially occurred through the improvement of the hepatic metabolism and inflammation.

The hallmark of NAFLD is abnormal triglyceride accumulation in the cytoplasm of hepatocytes, which results from an imbalance between lipid acquisition and lipid disposal. In NAFLD liver, uptake of lipid (Koonen et al., 2007; Zhu et al., 2011; Wilson et al., 2016) and *de novo* lipogenesis (Diraison et al., 2003; Lambert et al., 2014) are commonly enhanced. Lipid export *via* VLDL diminishes with NAFLD severity and ulteriorly results in hepatic lipid overload (Higuchi et al., 2011). Studies in fatty acid oxidation have yielded mixed results, but more evidence believes in increased rates of fatty acid oxidation in NAFLD (Sanyal et al., 2001; Begriche et al., 2013), which may be an adaptive response attempting to reduce the lipid overload (but usually not sufficient). The transcriptome analysis in this study highlighted the metabolic reprogramming events after PPC supplementation, which includes cholesterol biosynthesis, uptake of lipids, lipid synthesis, lipolysis, lipid export, and fatty acid oxidation. Coinciding with the transcriptome analysis, serum TG, LDL, AST, and ALT decreased, indicating PPC improved liver function in HFD mice. The metabolic reprogramming events are mainly manifested by changes in genes responsible for the rate-limiting steps. In HFD mice, enhanced hepatic lipid uptake mediated by fatty acid transport proteins (Fatp), cluster of differentiation 36 (CD36) is a typical pathophysiological change that contribute to NAFLD (Zhu et al., 2011). Meanwhile, silencing of Fatp2 and Fatp5 can improve NAFLD induced by HFD (Doege et al., 2006, 2008; Falcon et al., 2010). Acetyl coenzyme A carboxylase 1 (ACC1) and fatty acid synthase (FAS) are key enzymes in adipose synthesis, which catalyze malonyl-CoA and acetyl-CoA to generate palmitic acid (Maier et al., 2010; Kawano and Cohen, 2013). Sterol regulatory element-binding protein 1c (SREBP1c) is considered to be a master regulator that promotes the expression of lipogenic genes, including FAS and ACC1 (Horton et al., 2002). Carnitine palmitoyl transferase 1 (CPT1), the key enzyme in fatty acid oxidation, helps fatty acyl-CoAs transport across the outer mitochondrial membrane (McGarry and Foster, 1980; Kawano and Cohen, 2013). Activation of PPARα induces the transcription of genes related to fatty acid oxidation in organelles (e.g., ACOX), thereby promoting fatty acid oxidation (Fan et al., 1998; Nassir and Ibdah, 2014). ApoB 100, a long polypeptide facilitated by microsomal triglyceride transfer protein (MTTP), helps stabilize VLDL. Consequently, apoB 100 and MTTP are key components in hepatic lipid secretion (Hussain et al., 2003). In this study, PPC effectively repressed the expression of genes related to uptake of lipids and cholesterol synthesis. Meanwhile, genes related to fatty acid oxidation and lipid export were significantly upregulated. The results suggested that PPC alleviates the hepatic lipid overload and remodels hepatic lipid homeostasis in HFD mice. Interestingly, PPC was also observed to have a similar expression profile of genes related to lipid deposition and metabolic inflammation in the livers of low-fat diet-fed mice.

In recent years, emerging evidence points toward metabolic inflammation mediated by macrophages as a key process that contributes to the development of NAFLD. In HFD-induced NAFLD, macrophages are activated by palmitic acid and lipopolysaccharide (LPS) and produce inflammatory cytokines and chemokines, which affect β cell proliferation and function (Ying et al., 2019). As a result, insulin resistance is causally linked to macrophages in mice with diet-induced obesity (Wentworth et al., 2010). A recent study indicated that reduction of hepatic Ccr2^+^ monocyte-derived macrophages could result in improvement of glucose tolerance and hepatic triglyceride levels (Krenkel et al., 2018). Our results revealed a decrease in F4/80 and CD11c positive cells along with an increase in CD206 positive cells in liver after PPC supplementation, suggesting that although PPC increased the number of hepatic macrophages in HFD mice, the phenotype of these cells was anti-inflammatory type. Consistently, elevated expression of anti-inflammatory macrophage-related genes was observed after PPC supplementation. In our previous study, we also found that PPC inhibited pro-inflammatory macrophage polarization and improved LPS-induced inflammation in macrophages, and Toll-like receptors 2-mediated metabolic reprogramming could be an important mechanism (Feng et al., 2020). These findings revealed a novel anti-inflammatory mechanism of PPC.

Growing evidence suggests a link between deranged metabolism in the adipose tissue and development of NAFLD (Virtue and Vidal-Puig, 2010; Rosso et al., 2019; Azzu et al., 2020). In obesity, adipose tissue ceases to store energy efficiently and lipids begin to accumulate in other tissues including liver. Subsequently, ectopic lipid accumulation in hepatic cells results in NAFLD (Virtue and Vidal-Puig, 2010). On the other hand, recent studies highlight the role of adipose tissue macrophages and secretion of proinflammatory cytokines in the pathogenesis of NAFLD. In HFD mice, adipose tissue macrophage activation and pro-inflammatory gene expression preceded the development of inflammation in the liver (Stanton et al., 2011). Subcutaneous adipose tissue from patients with NAFLD was also reported to have an increased expression of genes that regulate inflammation (du Plessis et al., 2015). In this study, we found lower mass of subcutaneous adipose tissue alone with shrunken adipocytes after PPC treatment. Meanwhile, PPC modulated the gene expression of lipid synthesis and fatty acid oxidation and downregulated the expression of proinflammatory cytokines. Collectively, the findings indicated that PPC could promote lipolysis and mitigate inflammation in adipose tissue, which may have an improvement effect on NAFLD.

## Conclusion

**FIGURE 9:**
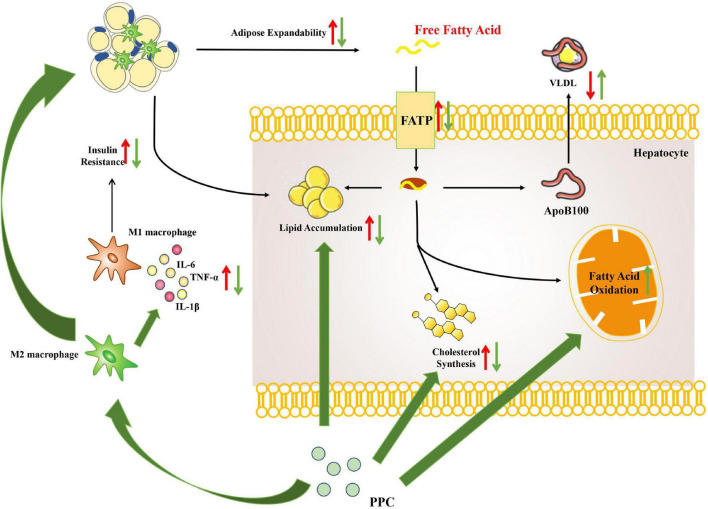
The overview of hepatic metabolic and inflammatory reprogramming in HFD mice after PPC intervention. HFD resulted in abnormal lipid accumulation and activated pro-inflammatory macrophage (red arrows), while PPC prevented the pathology of NAFLD via remodeling metabolism and inflammation (green arrows).

This study demonstrated that PPC improves HFD induced obesity and metabolic disorder. Moreover, the hepatoprotective effect of PPC on HFD induced hepatic steatosis was verified. Our results revealed that PPC reprograms the lipid metabolism and alleviates the metabolic inflammation (Figure 9). The findings provide novel insight into the amelioration of PPC on chronic liver diseases and offer beneficial instruction on clinical medication.

## Data Availability Statement

The datasets presented in this study can be found in online repositories. The names of the repository/repositories and accession number(s) can be found below: https://www.ncbi.nlm.nih.gov/sra/PRJNA752838.

## Ethics Statement

The animal study was reviewed and approved by the Laboratory Animal Welfare and Ethics Committee (LAWEC) of Xuzhou Medical University [Xuzhou, China, SCXK (Su) 2015-0009].

## Author Contributions

WP, QL, and XY conceived and designed the experiments. TF, MZ, JZ, PJ, MD, DX, and JW performed the experiments. WP, YL, TF, and MD analyzed the data. WP, FS, and QL contributed to reagents, materials, and analysis tools. WP, YL, QL, and XY wrote the manuscript. All authors contributed to the article and approved the submitted version.

## Conflict of Interest

The authors declare that the research was conducted in the absence of any commercial or financial relationships that could be construed as a potential conflict of interest.

## Publisher’s Note

All claims expressed in this article are solely those of the authors and do not necessarily represent those of their affiliated organizations, or those of the publisher, the editors and the reviewers. Any product that may be evaluated in this article, or claim that may be made by its manufacturer, is not guaranteed or endorsed by the publisher.

## Footnotes

1
http://powerandsamplesize.com/Calculators/Compare-k-Means/1-Way-ANOVA-Pairwise

2
https://imagej.net/imaging/particle-analysis

## References

[B1] Altschul S. F. Madden T. L. Schäffer A. A. Zhang J. Zhang Z. Miller W. (1997). Gapped BLAST and PSI-BLAST: a new generation of protein database search programs. *Nucleic Acids Res.* 25 3389–3402. 10.1093/nar/25.17.3389 9254694 PMC146917

[B2] Azzu V. Vacca M. Virtue S. Allison M. Vidal-Puig A. (2020). Adipose tissue-liver cross talk in the control of whole-body metabolism: implications in nonalcoholic fatty liver disease. *Gastroenterology* 158 1899–1912. 10.1053/j.gastro.2019.12.054 32061598

[B3] Bagchi D. P. MacDougald O. A. (2019). Identification and dissection of diverse mouse adipose depots. *J. Vis. Exp.* 149:10.3791/59499. 10.3791/59499 31355801 PMC7017470

[B4] Begriche K. Massart J. Robin M. A. Bonnet F. Fromenty B. (2013). Mitochondrial adaptations and dysfunctions in nonalcoholic fatty liver disease. *Hepatology* 58 1497–1507. 10.1002/hep.26226 23299992

[B5] Brunt E. M. (2010). Pathology of nonalcoholic fatty liver disease. *Nat. Rev. Gastroenterol. Hepatol.* 7 195–203. 10.1038/nrgastro.2010.21 20195271

[B6] Buzzetti E. Pinzani M. Tsochatzis E. A. (2016). The multiple-hit pathogenesis of non-alcoholic fatty liver disease (NAFLD). *Metabolism* 65 1038–1048. 10.1016/j.metabol.2015.12.012 26823198

[B7] Cao M. Li X. Zhang B. Han S. Yang Y. Zhou B. (2016). The effect of polyene phosphatidyl choline intervention on nonalcoholic steatohepatitis and related mechanism. *Am. J. Transl. Res.* 8 2325–2330. 27347340 PMC4891445

[B8] Cardiff R. D. Miller C. H. Munn R. J. (2014). Manual hematoxylin and eosin staining of mouse tissue sections. *Cold Spring Harb. Protoc.* 2014 655–658. 10.1101/pdb.prot073411 24890205

[B9] Dietrich P. Hellerbrand C. (2014). Non-alcoholic fatty liver disease, obesity and the metabolic syndrome. *Best Pract. Res. Clin. Gastroenterol.* 28 637–653. 10.1016/j.bpg.2014.07.008 25194181

[B10] Diraison F. Moulin P. Beylot M. (2003). Contribution of hepatic de novo lipogenesis and reesterification of plasma non esterified fatty acids to plasma triglyceride synthesis during non-alcoholic fatty liver disease. *Diabetes Metab.* 29 478–485. 10.1016/s1262-3636(07)70061-7 14631324

[B11] Doege H. Baillie R. A. Ortegon A. M. Tsang B. Wu Q. Punreddy S. (2006). Targeted deletion of FATP5 reveals multiple functions in liver metabolism: alterations in hepatic lipid homeostasis. *Gastroenterology* 130 1245–1258. 10.1053/j.gastro.2006.02.006 16618416

[B12] Doege H. Grimm D. Falcon A. Tsang B. Storm T. A. Xu H. (2008). Silencing of hepatic fatty acid transporter protein 5 *in vivo* reverses diet-induced non-alcoholic fatty liver disease and improves hyperglycemia. *J. Biol. Chem.* 283 22186–22192. 10.1074/jbc.M803510200 18524776 PMC2494916

[B13] du Plessis J. van Pelt J. Korf H. Mathieu C. van der Schueren B. Lannoo M. (2015). Association of adipose tissue inflammation with histologic severity of nonalcoholic fatty liver disease. *Gastroenterology* 149 635–48.e14. 10.1053/j.gastro.2015.05.044 26028579

[B14] Falcon A. Doege H. Fluitt A. Tsang B. Watson N. Kay M. A. (2010). FATP2 is a hepatic fatty acid transporter and peroxisomal very long-chain acyl-CoA synthetase. *Am. J. Physiol. Endocrinol. Metab.* 299 E384–E393. 10.1152/ajpendo.00226.2010 20530735 PMC2944282

[B15] Fan C. Y. Pan J. Usuda N. Yeldandi A. V. Rao M. S. Reddy J. K. (1998). Steatohepatitis, spontaneous peroxisome proliferation and liver tumors in mice lacking peroxisomal fatty acyl-CoA oxidase. *J. Biol. Chem.* 273 15639–15645. 10.1074/jbc.273.25.15639 9624157

[B16] Feng T. T. Yang X. Y. Hao S. S. Sun F. F. Huang Y. Lin Q. S. (2020). TLR-2-mediated metabolic reprogramming participates in polyene phosphatidylcholine-mediated inhibition of M1 macrophage polarization. *Immunol. Res.* 68 28–38. 10.1007/s12026-020-09125-9 32248343

[B17] Fischer A. H. Jacobson K. A. Rose J. Zeller R. (2008). Hematoxylin and eosin staining of tissue and cell sections. *CSH Protoc.* 2008:db.rot4986. 10.1101/pdb.prot4986 21356829

[B18] Han Y. H. Kim H. J. Na H. Nam M. W. Kim J. Y. Kim J. S. (2017). RORα induces KLF4-mediated M2 polarization in the liver macrophages that protect against nonalcoholic steatohepatitis. *Cell Rep.* 20 124–135. 10.1016/j.celrep.2017.06.017 28683306

[B19] Higuchi N. Kato M. Tanaka M. Miyazaki M. Takao S. Kohjima M. (2011). Effects of insulin resistance and hepatic lipid accumulation on hepatic mRNA expression levels of apoB, MTP and L-FABP in non-alcoholic fatty liver disease. experimental and therapeutic. *Medicine* 2 1077–1081. 10.3892/etm.2011.328 22977624 PMC3440820

[B20] Horton J. D. Goldstein J. L. Brown M. S. (2002). SREBPs: activators of the complete program of cholesterol and fatty acid synthesis in the liver. *J. Clin. Invest.* 109 1125–1131. 10.1172/JCI15593 11994399 PMC150968

[B21] Huang W. Metlakunta A. Dedousis N. Zhang P. Sipula I. Dube J. J. (2010). Depletion of liver Kupffer cells prevents the development of diet-induced hepatic steatosis and insulin resistance. *Diabetes* 59 347–357. 10.2337/db09-0016 19934001 PMC2809951

[B22] Hussain M. M. Shi J. Dreizen P. (2003). Microsomal triglyceride transfer protein and its role in apoB-lipoprotein assembly. *J. Lipid Res.* 44 22–32. 10.1194/jlr.r200014-jlr200 12518019

[B23] Kawano Y. Cohen D. E. (2013). Mechanisms of hepatic triglyceride accumulation in non-alcoholic fatty liver disease. *J. Gastroenterol.* 48 434–441. 10.1007/s00535-013-0758-5 23397118 PMC3633701

[B24] Kim D. Choi S. Y. Park E. H. Lee W. Kang J. H. Kim W. (2012). Nonalcoholic fatty liver disease is associated with coronary artery calcification. *Hepatology* 56 605–613. 10.1002/hep.25593 22271511 PMC3830979

[B25] Koonen D. P. Jacobs R. L. Febbraio M. Young M. E. Soltys C. L. Ong H. (2007). Increased hepatic CD36 expression contributes to dyslipidemia associated with diet-induced obesity. *Diabetes* 56 2863–2871. 10.2337/db07-0907 17728375

[B26] Krenkel O. Puengel T. Govaere O. Abdallah A. T. Mossanen J. C. Kohlhepp M. (2018). Therapeutic inhibition of inflammatory monocyte recruitment reduces steatohepatitis and liver fibrosis. *Hepatology* 67 1270–1283. 10.1002/hep.29544 28940700

[B27] Krenkel O. Tacke F. (2017). Macrophages in nonalcoholic fatty liver disease: a role model of pathogenic immunometabolism. *Semin. Liver Dis.* 37 189–197. 10.1055/s-0037-1604480 28847030

[B28] Lambert J. E. Ramos-Roman M. A. Browning J. D. Parks E. J. (2014). Increased de novo lipogenesis is a distinct characteristic of individuals with nonalcoholic fatty liver disease. *Gastroenterology* 146 726–735. 10.1053/j.gastro.2013.11.049 24316260 PMC6276362

[B29] Li C. Yang D. Cao X. Wang F. Jiang H. Guo H. (2016). LFG-500, a newly synthesized flavonoid, attenuates lipopolysaccharide-induced acute lung injury and inflammation in mice. *Biochem. Pharmacol.* 113 57–69. 10.1016/j.bcp.2016.05.007 27206337

[B30] Livak K. J. Schmittgen T. D. (2001). Analysis of relative gene expression data using real-time quantitative PCR and the 2(-Delta Delta C(T)) method. *Methods* 25 402–408. 10.1006/meth.2001.1262 11846609

[B31] Lv Y. Gao X. Luo Y. Fan W. Shen T. Ding C. (2019). Apigenin ameliorates HFD-induced NAFLD through regulation of the XO/NLRP3 pathways. *J. Nutr. Biochem.* 71 110–121. 10.1016/j.jnutbio.2019.05.015 31325892

[B32] Maev I. V. Samsonov A. A. Palgova L. K. Pavlov C. S. Vovk E. I. Shirokova E. N. (2020). Effectiveness of phosphatidylcholine in alleviating steatosis in patients with non-alcoholic fatty liver disease and cardiometabolic comorbidities (MANPOWER study). *BMJ Open Gastroenterol.* 7:e000341. 10.1136/bmjgast-2019-000341 32095253 PMC7011021

[B33] Maier T. Leibundgut M. Boehringer D. Ban N. (2010). Structure and function of eukaryotic fatty acid synthases. *Q. Rev. Biophys.* 43 373–422. 10.1017/S0033583510000156 20731893

[B34] Manne V. Handa P. Kowdley K. V. (2018). Pathophysiology of nonalcoholic fatty liver disease/nonalcoholic steatohepatitis. *Clin. Liver Dis.* 22 23–37. 10.1016/j.cld.2017.08.007 29128059

[B35] Matthews D. R. Hosker J. P. Rudenski A. S. Naylor B. A. Treacher D. F. Turner R. C. (1985). Homeostasis model assessment: insulin resistance and beta-cell function from fasting plasma glucose and insulin concentrations in man. *Diabetologia* 28 412–419. 10.1007/BF00280883 3899825

[B36] McGarry J. D. Foster D. W. (1980). Regulation of hepatic fatty acid oxidation and ketone body production. *Annu. Rev. Biochem.* 49 395–420. 10.1146/annurev.bi.49.070180.002143 6157353

[B37] Mi L. J. Mak K. M. Lieber C. S. (2000). Attenuation of alcohol-induced apoptosis of hepatocytes in rat livers by polyenylphosphatidylcholine (PPC). *Alcohol. Clin. Exp. Res.* 24 207–212. 10.1111/j.1530-0277.2000.tb04592.x 10698373

[B38] Musso G. Cassader M. Cohney S. Pinach S. Saba F. Gambino R. (2015). Emerging liver-kidney interactions in nonalcoholic fatty liver disease. *Trends Mol. Med.* 21 645–662. 10.1016/j.molmed.2015.08.005 26432021

[B39] Nagy C. Einwallner E. (2018). Study of *in vivo* glucose metabolism in high-fat diet-fed mice using oral glucose tolerance test (OGTT) and insulin tolerance test (ITT). *J. Vis. Exp.* 131:56672. 10.3791/56672 29364280 PMC5908452

[B40] Nassir F. Ibdah J. A. (2014). Role of mitochondria in nonalcoholic fatty liver disease. *Int. J. Mol. Sci.* 15 8713–8742. 10.3390/ijms15058713 24837835 PMC4057755

[B41] Okiyama W. Tanaka N. Nakajima T. Tanaka E. Kiyosawa K. Gonzalez F. J. (2009). Polyenephosphatidylcholine prevents alcoholic liver disease in PPARalpha-null mice through attenuation of increases in oxidative stress. *J. Hepatol.* 50 1236–1246. 10.1016/j.jhep.2009.01.025 19398233 PMC2809859

[B42] Pan W. Hao W. T. Xu H. W. Qin S. P. Li X. Y. Liu X. M. (2017). Polyene phosphatidylcholine inhibited the inflammatory response in LPS-stimulated macrophages and ameliorated the adjuvant-induced rat arthritis. *Am. J. Transl. Res.* 9 4206–4216. 28979694 PMC5622263

[B43] Park S. H. Kim D. J. Plank L. D. (2020). Association of grip strength with non-alcoholic fatty liver disease: investigation of the roles of insulin resistance and inflammation as mediators. *Eur. J. Clin. Nutr.* 74 1401–1409. 10.1038/s41430-020-0591-x 32152511

[B44] Peverill W. Powell L. W. Skoien R. (2014). Evolving concepts in the pathogenesis of NASH: beyond steatosis and inflammation. *Int. J. Mol. Sci.* 15 8591–8638. 10.3390/ijms15058591 24830559 PMC4057750

[B45] Robinson M. D. McCarthy D. J. Smyth G. K. (2010). edgeR: a Bioconductor package for differential expression analysis of digital gene expression data. *Bioinformatics* 26 139–140. 10.1093/bioinformatics/btp616 19910308 PMC2796818

[B46] Robinson M. D. Oshlack A. (2010). A scaling normalization method for differential expression analysis of RNA-seq data. *Genome Biol.* 11:R25. 10.1186/gb-2010-11-3-r25 20196867 PMC2864565

[B47] Robinson M. D. Smyth G. K. (2008). Small-sample estimation of negative binomial dispersion, with applications to SAGE data. *Biostatistics* 9 321–332. 10.1093/biostatistics/kxm030 17728317

[B48] Rosso C. Kazankov K. Younes R. Esmaili S. Marietti M. Sacco M. (2019). Crosstalk between adipose tissue insulin resistance and liver macrophages in non-alcoholic fatty liver disease. *J. Hepatol.* 71 1012–1021. 10.1016/j.jhep.2019.06.031 31301321

[B49] Ruhl C. E. Everhart J. E. (2004). Epidemiology of nonalcoholic fatty liver. *Clin. Liver Dis.* 8 501–519. 10.1016/j.cld.2004.04.008 15331060

[B50] Sanyal A. J. Campbell-Sargent C. Mirshahi F. Rizzo W. B. Contos M. J. Sterling R. K. (2001). Nonalcoholic steatohepatitis: association of insulin resistance and mitochondrial abnormalities. *Gastroenterology* 120 1183–1192. 10.1053/gast.2001.23256 11266382

[B51] Seebacher F. Zeigerer A. Kory N. Krahmer N. (2020). Hepatic lipid droplet homeostasis and fatty liver disease. *Semin. Cell Dev. Biol.* 108 72–81. 10.1016/j.semcdb.2020.04.011 32444289

[B52] Stanton M. C. Chen S. C. Jackson J. V. Rojas-Triana A. Kinsley D. Cui L. (2011). Inflammatory Signals shift from adipose to liver during high fat feeding and influence the development of steatohepatitis in mice. *J. Inflammation* 8:8. 10.1186/1476-9255-8-8 21410952 PMC3070617

[B53] Virtue S. Vidal-Puig A. (2010). Adipose tissue expandability, lipotoxicity and the metabolic syndrome–an allostatic perspective. *Biochim. Biophys. Acta* 1801 338–349. 10.1016/j.bbalip.2009.12.006 20056169

[B54] Wan J. Benkdane M. Teixeira-Clerc F. Bonnafous S. Louvet A. Lafdil F. (2014). M2 Kupffer cells promote M1 Kupffer cell apoptosis: a protective mechanism against alcoholic and nonalcoholic fatty liver disease. *Hepatology* 59 130–142. 10.1002/hep.26607 23832548

[B55] Wentworth J. M. Naselli G. Brown W. A. Doyle L. Phipson B. Smyth G. K. (2010). Pro-inflammatory CD11c+CD206+ adipose tissue macrophages are associated with insulin resistance in human obesity. *Diabetes* 59 1648–1656. 10.2337/db09-0287 20357360 PMC2889764

[B56] Wilson C. G. Tran J. L. Erion D. M. Vera N. B. Febbraio M. Weiss E. J. (2016). Hepatocyte-specific disruption of CD36 attenuates fatty liver and improves insulin sensitivity in HFD-Fed mice. *Endocrinology* 157 570–585. 10.1210/en.2015-1866 26650570 PMC4733118

[B57] Ying W. Lee Y. S. Dong Y. Seidman J. S. Yang M. Isaac R. (2019). Expansion of islet-resident macrophages leads to inflammation affecting β cell proliferation and function in obesity. *Cell Metab.* 29 457–474.e5. 10.1016/j.cmet.2018.12.003 30595478 PMC6701710

[B58] Yki-Järvinen H. (2014). Non-alcoholic fatty liver disease as a cause and a consequence of metabolic syndrome. *Lancet Diabetes Endocrinol.* 2 901–910. 10.1016/S2213-8587(14)70032-4 24731669

[B59] Younossi Z. M. Marchesini G. Pinto-Cortez H. Petta S. (2019). Epidemiology of Nonalcoholic Fatty Liver Disease and Nonalcoholic Steatohepatitis: implications for Liver Transplantation. *Transplantation* 103 22–27. 10.1097/TP.0000000000002484 30335697

[B60] Zhu L. Baker S. S. Liu W. Tao M. H. Patel R. Nowak N. J. (2011). Lipid in the livers of adolescents with nonalcoholic steatohepatitis: combined effects of pathways on steatosis. *Metabolism* 60 1001–1011. 10.1016/j.metabol.2010.10.003 21075404

