# Supplementary material for: Polyene Phosphatidylcholine Ameliorates High Fat Diet-Induced Non-alcoholic Fatty Liver Disease via Remodeling Metabolism and Inflammation
Source: Front Physiol. 2022 Feb 28;13:810143. doi: 10.3389/fphys.2022.810143 (PMC8918669; doi:10.3389/fphys.2022.810143)
Supplement: Supplementary file 1 [file Data_Sheet_1.docx]

Supplementary Material

**Figure S1. The distribution of differentially expressed genes (DEGs).** (A) The correction heatmap of samples in different groups. (B) The venn diagram of DEGs. (C) The venn diagram of upregulated DEGs. (D) The venn diagram of downregulated DEGs.

**Figure S2. The transcriptome analysis of GO annotation.** Go annotation of DEGs between HFD+PPC and HFD with top 30 enrichment scores covering domains of biological processes (A), cellular components (B) and molecular functions (C).

**Figure** **S3. The transcriptome analysis of KEGG pathway.** (A) The bubble chart shows the top 30 terms of KEGG pathways of DEGs between HFD+PPC and HFD mice. (B) The column chart shows the top 10 terms of KEGG second class pathway of DEGs between HFD+PPC and HFD mice.

**Figure S4.** **PPC supplementation enhances lipolysis and alleviates inflammation in the epididymal adipose tissues of mice fed by HFD.** (A) Representative adipose tissue images of H&E staining. (B) The statistical results of epididymal adipocyte diameter. (C) The statistical results of epididymal adipocyte superficial area. (D) The expression of key enzymes in glucose metabolism. (E) The expression of key enzymes related to fatty acid metabolism. The scaleplate of the representative images is 50 μm. n=10 for each group. The differences were analyzed using ANOVA. Data represent means with SEM. The pound signs indicate statistically significant differences compared to the LC group. ^###^*P* < 0.001. Asterisks indicate statistically significant differences compared to the HFD group. **P* < 0.05, ***P* < 0.01.

**Figure S5.**  **PPC supplementation remodels the transcriptome profile of liver in the mice fed by LC**. (A) The number of DEGs. (B) The volcano plot shows the distributions of DEGs between LC and PPC mice. The x-axis indicates the fold change. red dots, upregulated; green dots, downregulated. (C) Go annotation of DEGs between LC and PPC with terms related to hepatic metabolism and inflammation. n=3 for each group.

**Figure S6. PPC modulates hepatic metabolism and immunity in LC fed mice.** (A)The bubble chart shows the dysregulated terms in three KEGG levels between LC and PPC mice (*P* <0.05). The larger the point, the more genes fall into this pathway and the yellower point means higher significance of enrichment. (B) The expression of genes in lipid metabolism and inflammation. n=3 for each group. The differences were analyzed using two tailed Student’s t-test. Data represent means with SEM. **P* < 0.05, ***P* < 0.01.

**Table S1. The primer sequences used in this study.**

**Table S2. Differentially expressed genes in the livers of HFD fed mice after PPC supplementation.**

**Table S3. Differentially expressed genes in the livers of LC fed mice after PPC supplementation.**

**Table S4. GO enrichment analysis of genes in the livers of HFD fed mice after PPC supplementation.**

**Table S5. KEGG pathways analysis of genes in the livers of HFD mice after PPC supplementation.**
