# Supplementary figures and images for: Polyene Phosphatidylcholine Ameliorates High Fat Diet-Induced Non-alcoholic Fatty Liver Disease via Remodeling Metabolism and Inflammation
Source: Front Physiol. 2022 Feb 28;13:810143. doi: 10.3389/fphys.2022.810143 (PMC8918669; doi:10.3389/fphys.2022.810143)

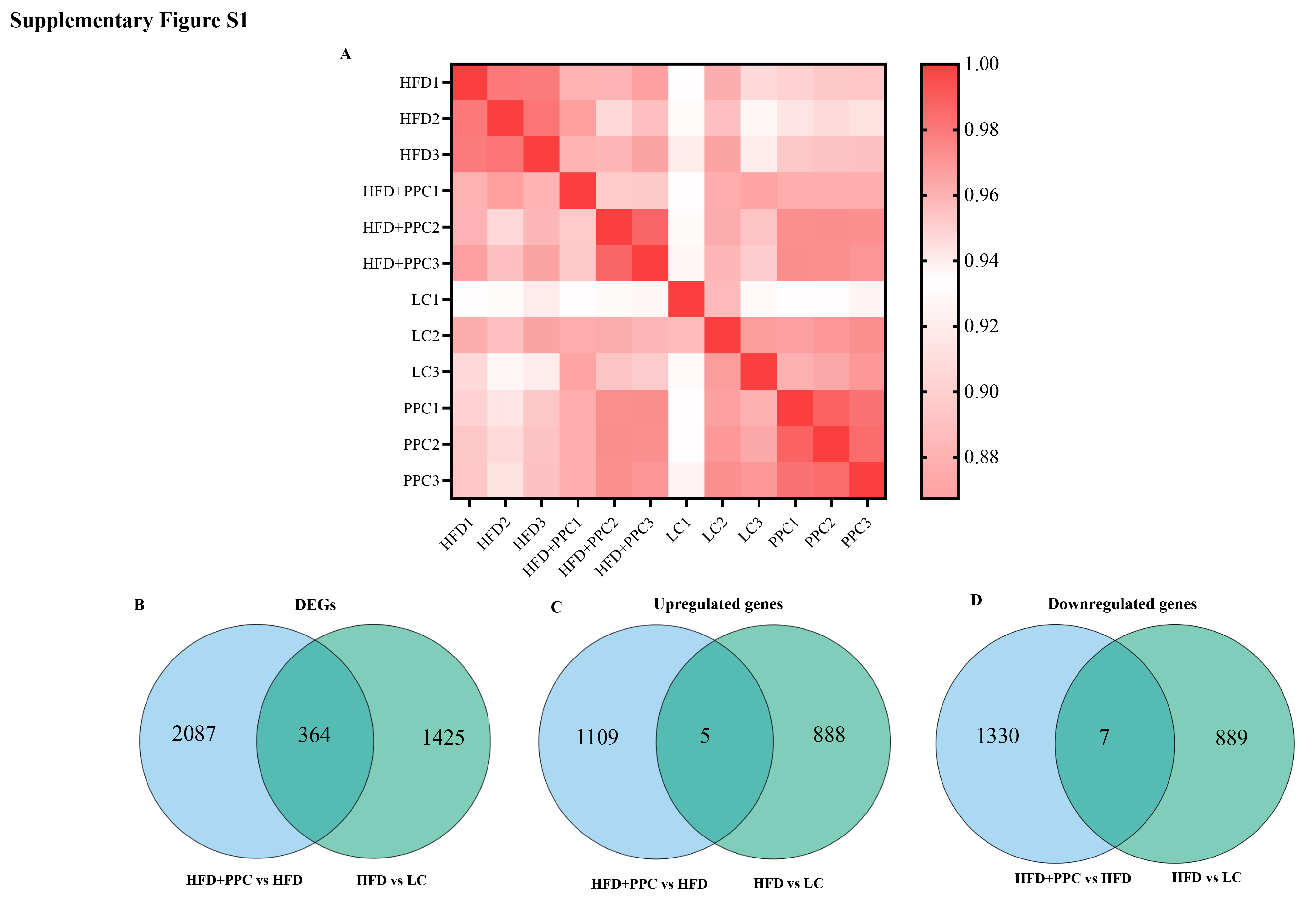

Supplement: Supplementary file 7 [file Image_1.jpg]

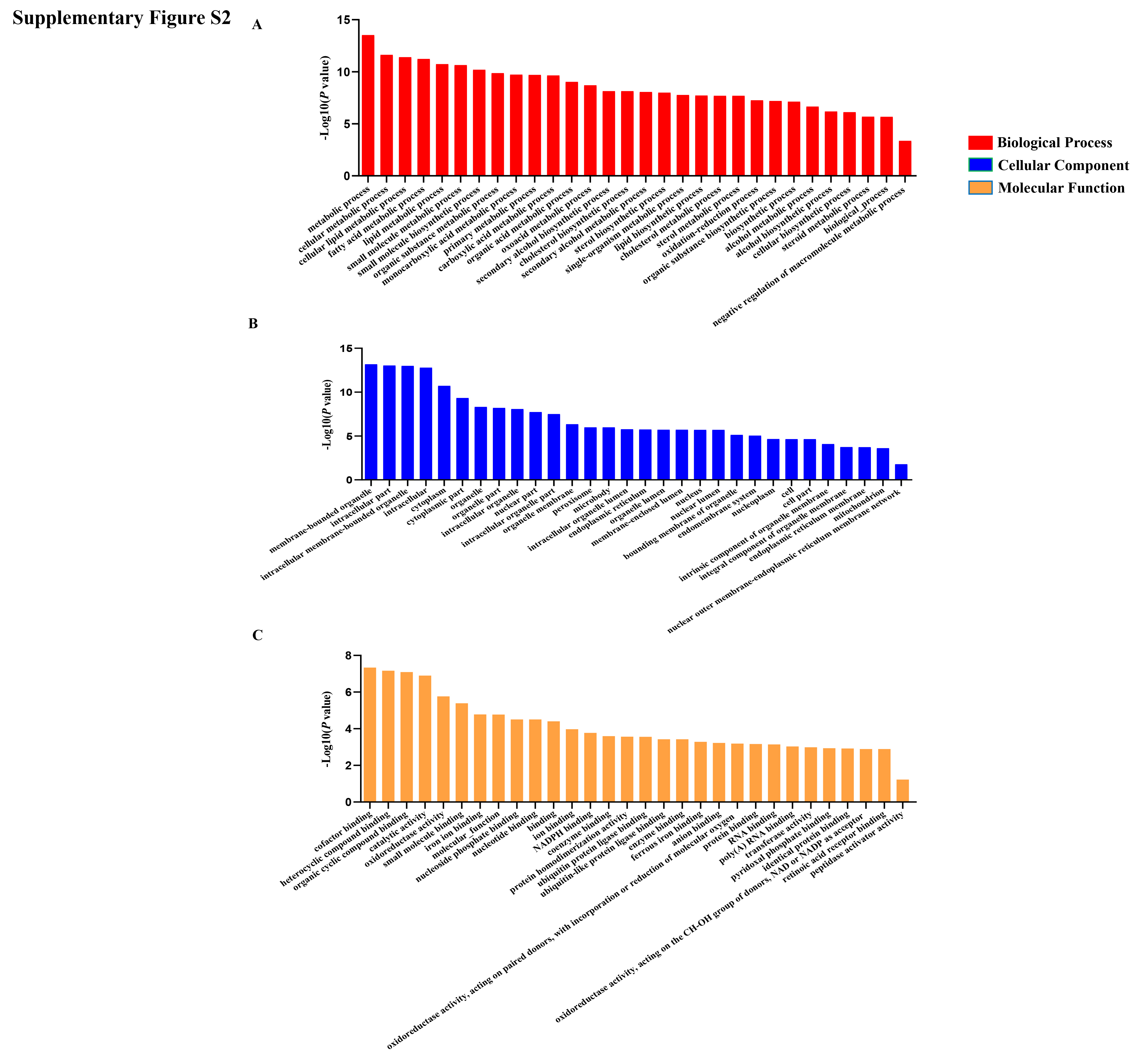

Supplement: Supplementary file 8 [file Image_2.jpg]

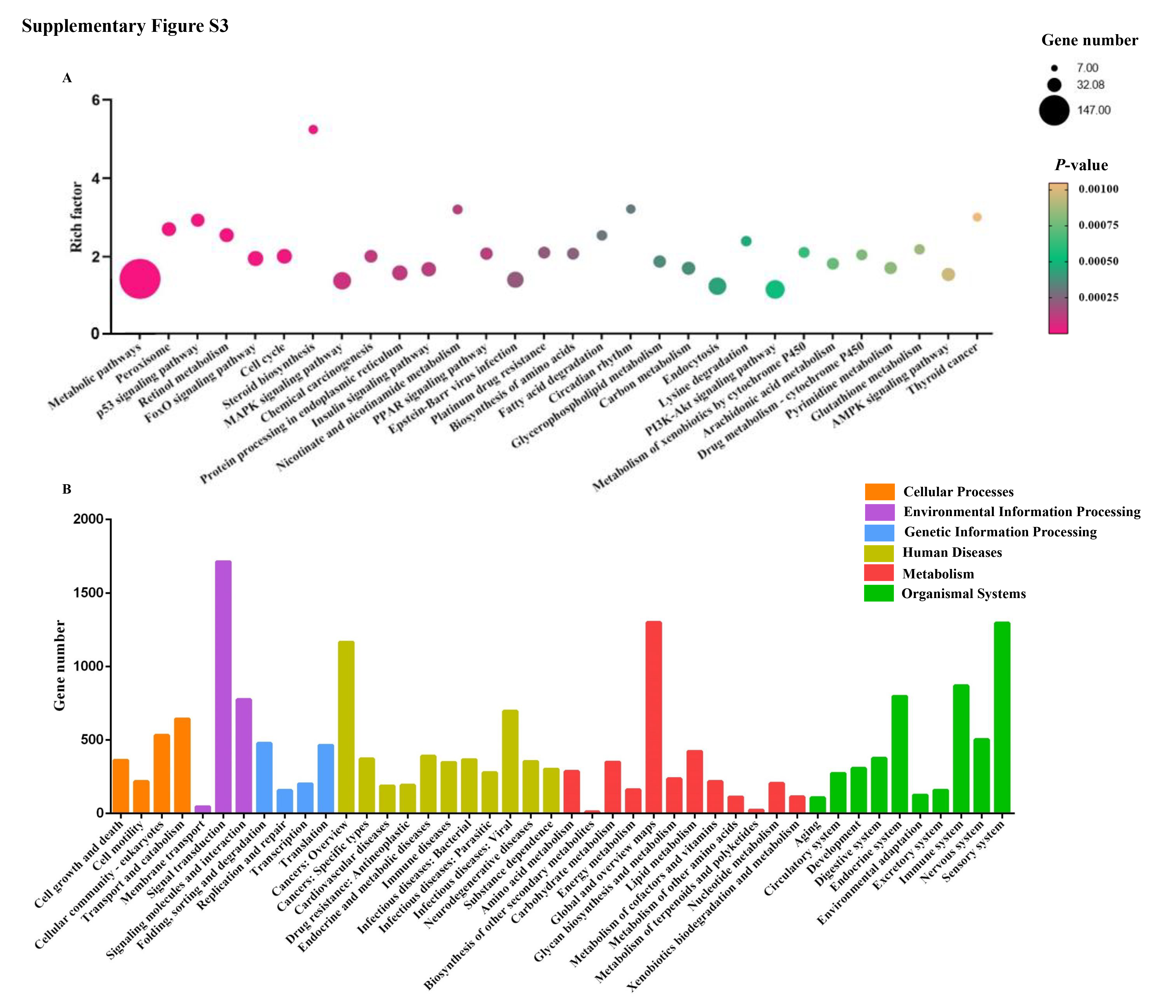

Supplement: Supplementary file 9 [file Image_3.jpg]

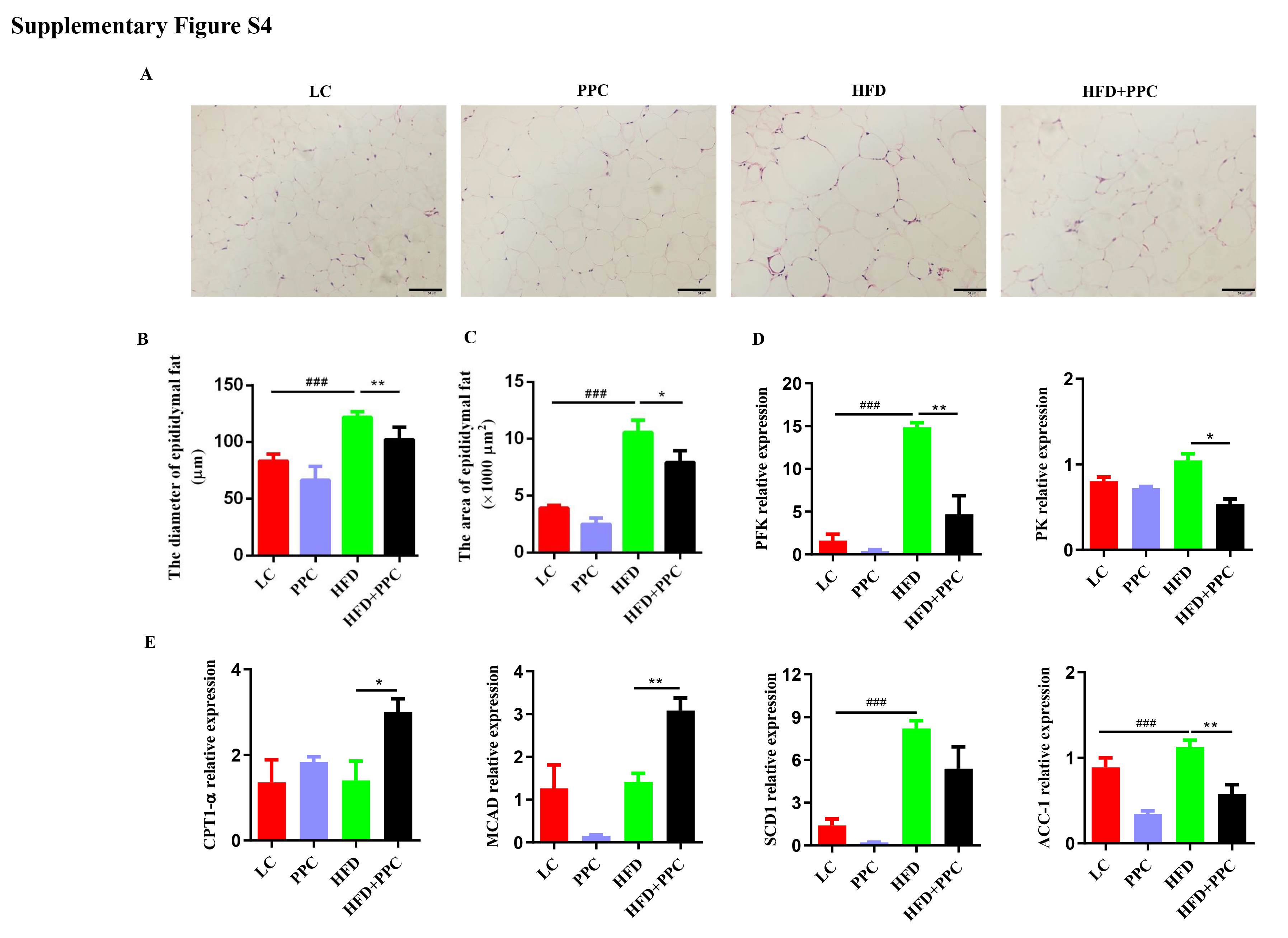

Supplement: Supplementary file 10 [file Image_4.jpg]

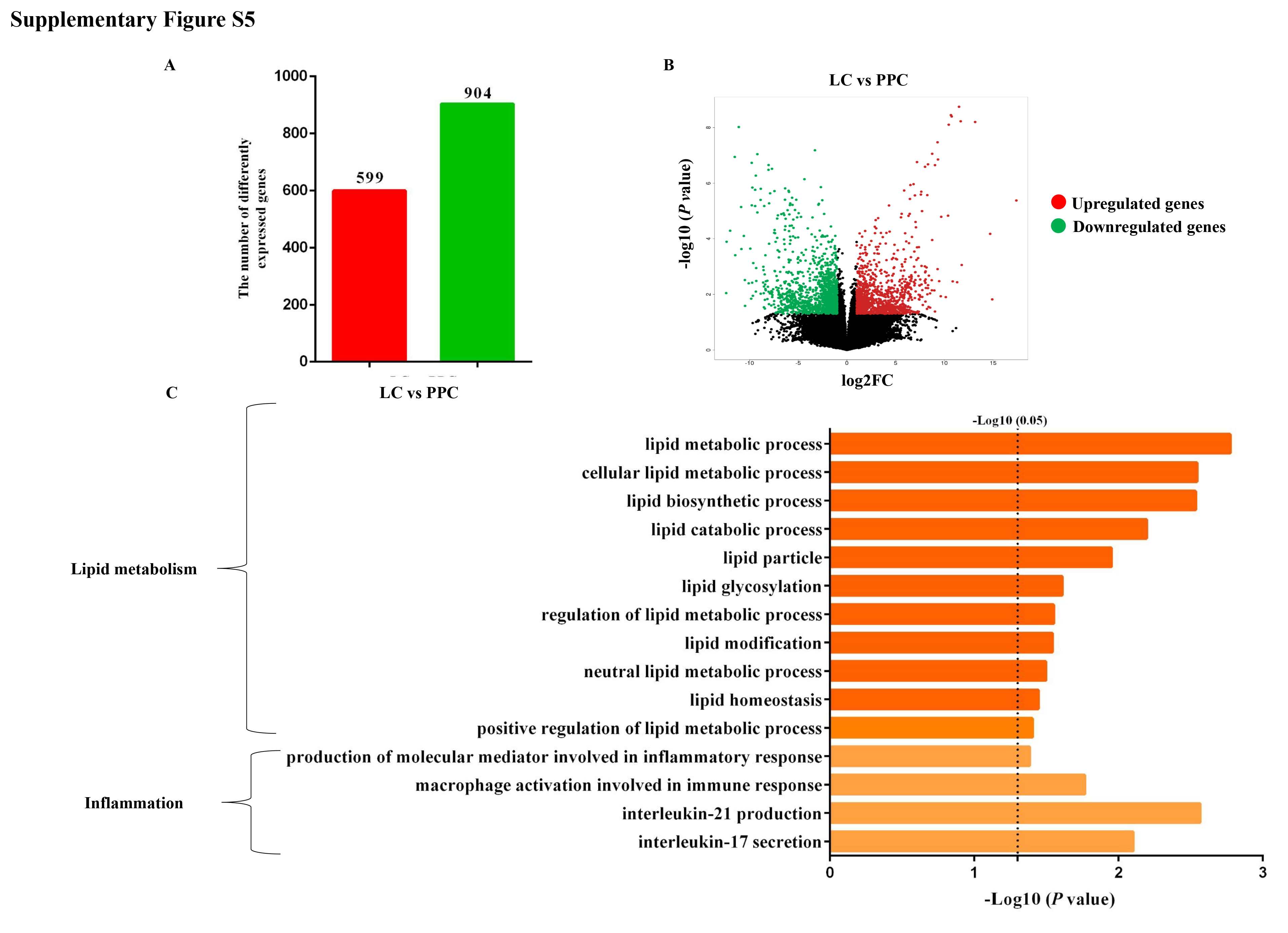

Supplement: Supplementary file 11 [file Image_5.jpg]

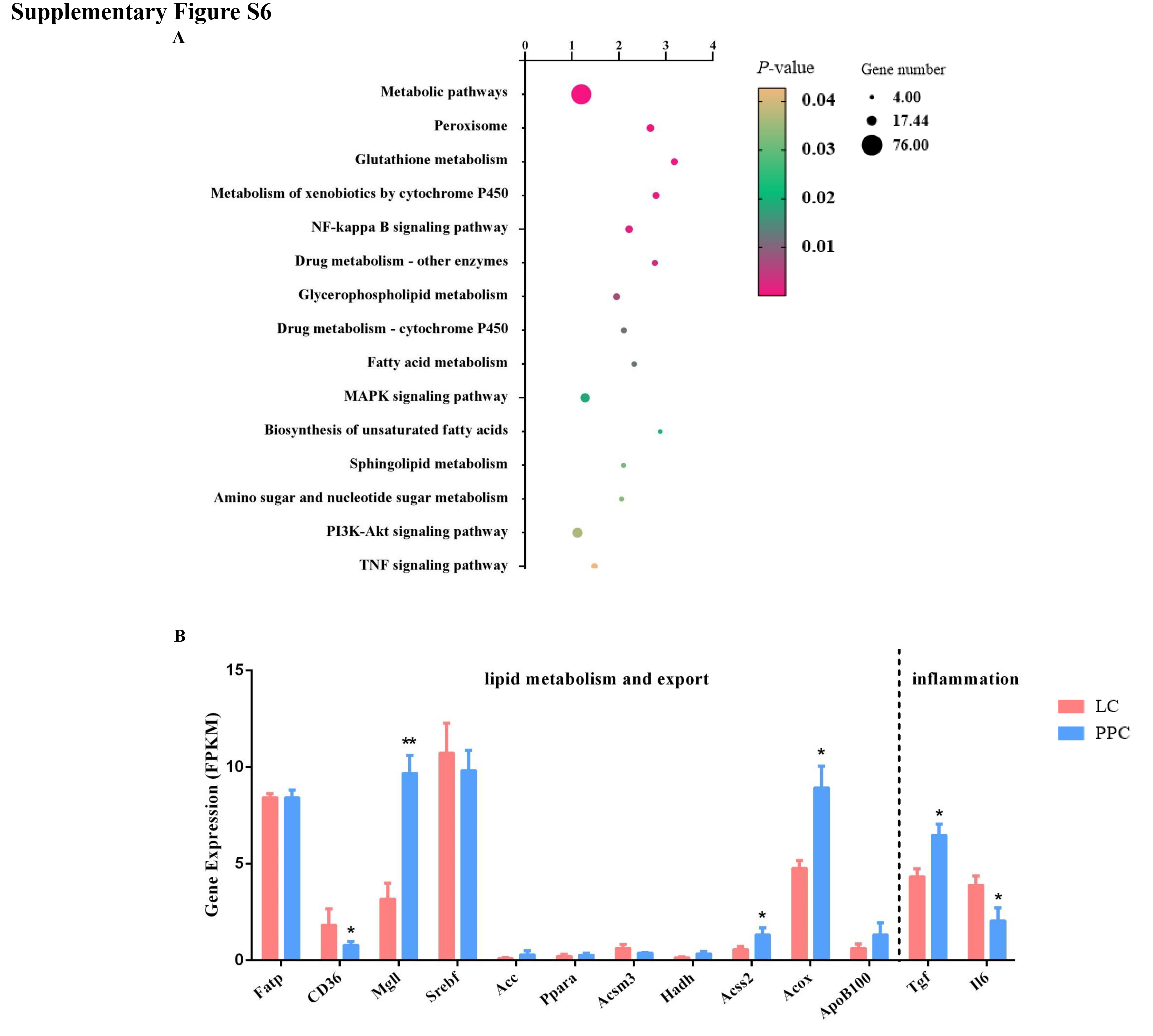

Supplement: Supplementary file 12 [file Image_6.jpg]
